# Increasing the Performance and Stability of Red-Light-Emitting Diodes Using Guanidinium Mixed-Cation Perovskite Nanocrystals

**DOI:** 10.1021/acs.chemmater.3c00269

**Published:** 2023-05-09

**Authors:** Patricio Serafini, Alexis Villanueva-Antolí, Samrat Das Adhikari, Sofia Masi, Rafael S. Sánchez, Jhonatan Rodriguez-Pereira, Bapi Pradhan, Johan Hofkens, Andrés F. Gualdrón-Reyes, Iván Mora-Seró

**Affiliations:** † Institute of Advanced Materials (INAM), Universitat Jaume I, Avenida de Vicent Sos Baynat, s/n, Castelló de la Plana, Castellón 12071, Spain; ‡ Center of Materials and Nanotechnologies, Faculty of Chemical Technology, University of Pardubice, 53002 Pardubice, Czech Republic; § Central European Institute of Technology, Brno University of Technology, 612 00 Brno, Czech Republic; ∥ Laboratory for Photochemistry and Spectroscopy, Molecular Imaging and Photonics, Department of Chemistry, Katholieke Universiteit Leuven, Celestijnenlaan 200F − bus 2404, B-3001 Heverlee, Belgium; ⊥ Facultad de Ciencias, Instituto de Ciencias Químicas, Isla Teja, Universidad Austral de Chile, 5090000 Valdivia, Chile

## Abstract

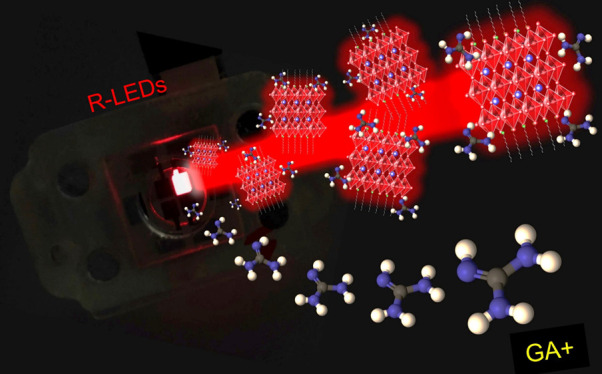

Halide perovskite nanocrystals (PNCs) exhibit growing attention in optoelectronics due to their fascinating color purity and improved intrinsic properties. However, structural defects emerging in PNCs progressively hinder the radiative recombination and carrier transfer dynamics, limiting the performance of light-emitting devices. In this work, we explored the introduction of guanidinium (GA^+^) during the synthesis of high-quality Cs_1–*x*_GA_*x*_PbI_3_ PNCs as a promising approach for the fabrication of efficient bright-red light-emitting diodes (R-LEDs). The substitution of Cs by 10 mol % GA allows the preparation of mixed-cation PNCs with PLQY up to 100% and long-term stability for 180 days, stored under air atmosphere and refrigerated condition (4 °C). Here, GA^+^ cations fill/replace Cs^+^ positions into the PNCs, compensating intrinsic defect sites and suppressing the nonradiative recombination pathway. LEDs fabricated with this optimum material show an external quantum efficiency (EQE) near to 19%, at an operational voltage of 5 V (50–100 cd/m^2^) and an operational half-time (*t*_50_) increased 67% respect CsPbI_3_ R-LEDs. Our findings show the possibility to compensate the deficiency through A-site cation addition during the material synthesis, obtaining less defective PNCs for efficient and stable optoelectronic devices.

## 1 Introduction

Halide perovskite nanocrystals (PNCs) have consolidated as one of the most promising photoactive materials^1^ due to their tunable emission wavelength,^2^ narrow photoluminescence (PL) bandwidth, low-cost processing, high color purity,^3,4^ PL quantum yield (PLQY) up to 100%, and versatile surface chemistry.^5^ These fascinating features have facilitated the fabrication of efficient light-emitting diodes (LEDs) with external quantum efficiencies (EQEs) >20%,^6,7^ for green and red emissions. However, the development of high-performance, bright, and stable red-emitting perovskite LEDs is more demanding than the case of their green counterparts. In the case of perovskite green LEDs, the highest EQE is ∼28.9%, with an operational lifetime of >30,000 h at 100 cd/m^2^ and a luminance (*L*) of 470,000 cd/m^2^.^8^ This *L* value is similar to the highest one achieved for this perovskite ∼591,197 cd/m^2^.^9^ However, for red LEDs (R-LEDs) based on CsPbI_3_, their highest *L* is significantly lower in comparison to green LEDs, with the record value of *L* = 10,170 cd/m^2^,^10^ maximum EQE ∼23%,^11^ and the longest operation lifetime of 317 h when measuring at 30 mA/cm^2^.^12^ This limitation in comparison with green LEDs is due mostly to the perovskite black α-phase stability of CsPbI_3_ that it is prone to suffer the deterioration of its PL properties and stability as a consequence of the fast α-to-δ phase transformation, limiting its use in long-term applications.^13,14^ Therefore, it is pivotal to find a facile and suitable strategy to prepare iodide PNCs with improved PL properties, which can provide better R-LEDs with parameters closer to their analogous green LEDs.

Diverse strategies have been studied to improve the stability and optical performance of red-emitting PNCs such as ligand surface passivation with capping ligands with better binding energy than the traditional oleic acid/oleylamine,^15,16^ modified synthetic routes for PNC growth and purification,^17^ and composition engineering.^18,19^ Beyond these alternatives, the A-site cation engineering has been studied to enhance the structural stability of the active layer and the device operation by slightly tilting the metal-halide octahedra to create a sustainable crystal strain.^20−24^

From a structural point of view, the ABX_3_ perovskite can adopt different crystalline phases depending on the size and interaction of the A cation located at the center of the unit cell, connected to the corner-sharing BX_6_ octahedra. The structural stability has been extensively evaluated using the Goldschmidt tolerance factor (*t*),^25^ defined as , where *r*_A_ is the ionic radius of the cations, *r*_B_ is the metal cation, and *r*_X_ are the halides, ‘*t*’ being a stable structure in the approximated range of 0.8 < *t* < 1.0.^26,27^ In this context, a cation such as Cs^+^ gives the tolerance factor too low to sustain a cubic perovskite structure at room temperature, resulting in a nonphotoactive nonperovskite δ-yellow phase with an orthorhombic structure. To overcome this issue, the A-site cation alloying is a useful tool to modulate the tolerance factor of the perovskite structure, being beneficial to control its optical features and chemical stability. Formamidinium (FA^+^) cation has been widely reported as one of the most suitable species to partially replace Cs^+^ into the CsPbI_3_ PNCs, hindering the α-to-δ phase transition at room temperature and stabilizing the black crystalline α-phase.^28,29^ Although the nanoconfinement of particle size and the A-site alloying extend the chemical durability of PNCs, the incorporation of FA^+^ displaces the PL emission of the CsPbI_3_ PNCs from red to infrared region spectra. This produces a displacement of the PL peak position away from the range 620–635 nm, which is the Rec. 2020 standard for pure red color used as the reference in industry for the fabrication of ultrahigh definition LCD displays. In addition, the synthesis of A-site-alloyed PNCs is still a challenge since the thermodynamics of the mixed-cation phases, crystal surface energy, and the stoichiometry of the precursors induce the formation of defective nanocrystals,^25^ which can produce PLQY far from unity and an eventual quenching of their PL properties. Under this premise, the incorporation of big size cations beyond the limit of the tolerance factor would be ideal to prepare mixed-cation PNCs with stable optical features and lesser defective structures. This fact can maximize the operational performance of LEDs.

In this work, we analyzed the incorporation of guanidinium (GA^+^) cation during the synthesis of colloidal mixed-cation Cs_1–*x*_GA_*x*_PbI_3_ PNCs with improved optical performance and a lowered defective structure, suitable for the fabrication of efficient bright perovskite R-LEDs. Considering that some works report that high ratios of GA^+^ result in the formation of 2D layered perovskites due to its big size,^30−32^ here, we were able to establish that the addition of a percentage of GA^+^ equal or lower than 30 mol % has been considered. This GA content was used to tailor the tolerance factor of CsPbI_3_ PNCs looking for a stable black α-cubic phase perovskite, without altering the initial crystalline phase of the host. Therefore, the presence of a 2D layered perovskite was not found. By replacing Cs by 10 mol % GA in the mixture reaction, we were able to obtain mixed-cation PNCs with 100% photoluminescence quantum yield (PLQY) and long-term stability of at least 180 days. In addition, we observed that GA^+^ species fill/replace the Cs^+^ positions and facilitate the passivation of iodide vacancies into the PNCs, maximizing the suppression of nonradiative carrier traps. Under these optimized synthesis conditions, bright perovskite R-LEDs with an external quantum efficiency (EQE) ∼19% were achieved. However, the R-LED performance decreases at higher GA^+^ contents, deducing that a high density of these species generates a steric hindrance on the PNC surface, restraining the compensation of defect sites. This work shows a facile synthetic route through the A-site cation tuning to prepare mixed-cation Cs_1–*x*_GA_*x*_PbI_3_ PNCs with enhanced intrinsic properties to fabricate high-performance and stable R-LEDs.

## 2 Results and Discussion

**Figure 1:**
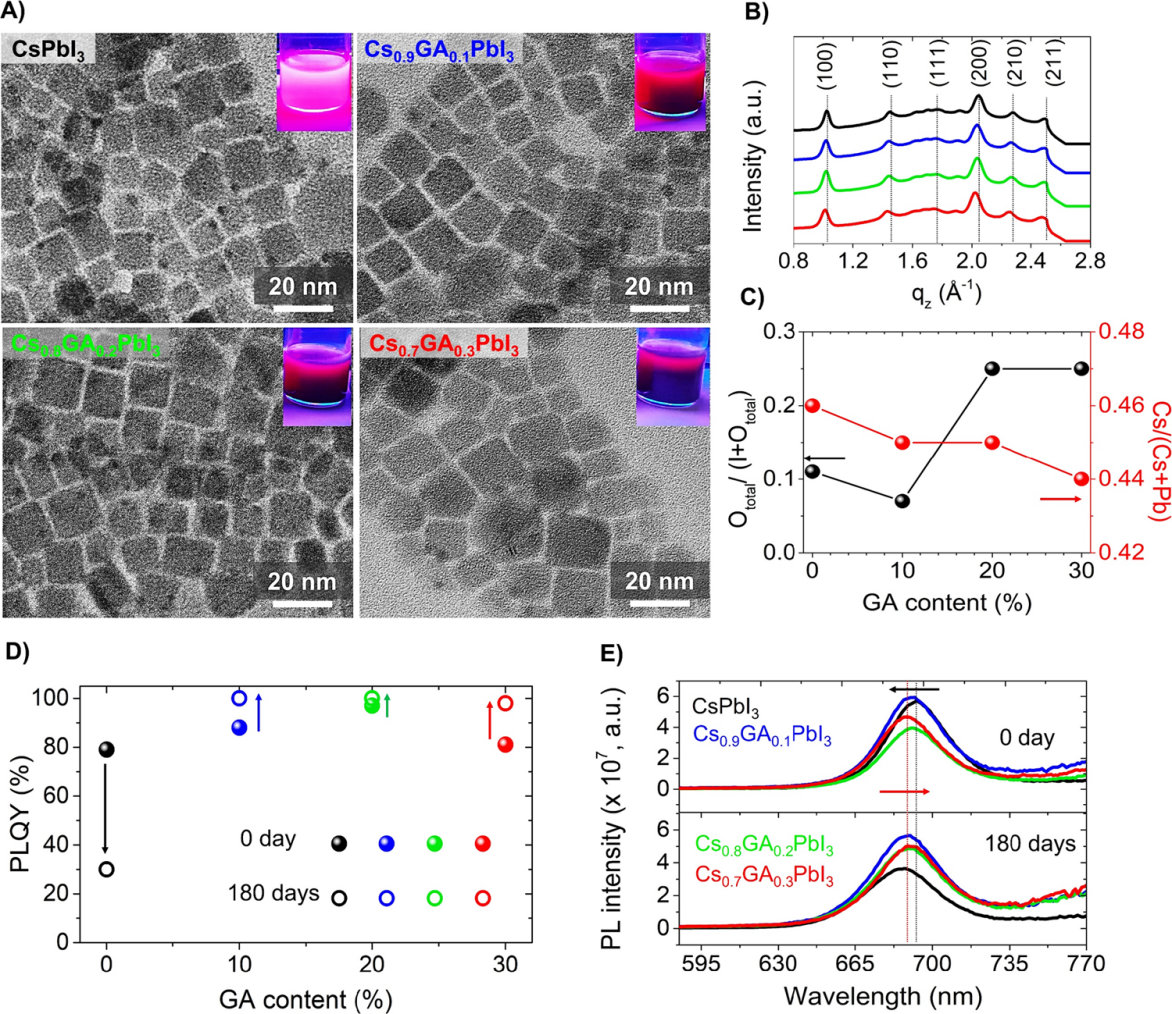
(A) TEM images of CsPbI_3_ and Cs_1–*x*_GA_*x*_PbI_3_ PNCs in the presence of 10, 20, and 30 mol % GA. Inset shows the corresponding SAED patterns. (B) Out-of-plane line cuts from the 2D GIWAXS patterns of CsPbI_3_ and Cs_1–*x*_GA_*x*_PbI_3_ PNC thin films. (C) Oxygen with respect to halide relation and Cs to metal cation relation. (D) PLQY and (E) PL spectra for each mol% GA for fresh (0 days) and aged samples (180 days).

Figure 1A shows the transmission electron microscopy (TEM) images of the mixed-cation Cs_1–*x*_GA_*x*_PbI_3_ PNCs, varying the GA content added during the material synthesis (*x* = 0.1, 0.2, and 0.3). In all the cases, Cs_1–*x*_GA_*x*_PbI_3_ PNCs exhibit a cubic morphology, with a slightly bigger average particle size and broader particle size distribution than that of pristine CsPbI_3_ PNCs (Figure S1). We associated this change to the modification of stoichiometry of the precursors into the mixture reaction by Cs-to-GA substitution, where the *instant capping* process is induced to favor the emergence of a smaller particle size. Here, low-density GA cations could also be acting as capping ligands, replacing/filling A-site cation vacancies as similar as bulky oleylammonium species, stabilizing the final product.^19^ However, the smaller ionic radius of GA^+^ than that of OLAm^+^ could allow GA introduction. Simultaneously, we suggest that the stoichiometry alteration during the synthesis by GA introduction facilitates the coarsening of PNCs, also producing bigger nanoparticles.^33^ In addition, no significant changes are observed in the selected area electron diffraction (SAED) patterns obtained from the TEM measurements, but the pattern definition enhances with GA addition compared to the pristine sample (Figure S2). This fact indicates that the presence of GA cations does not produce any change in the crystalline phase; on the contrary, the crystallinity of the GA-modified samples is improved. On the other hand, synchrotron-assisted grazing incidence wide angle X-ray scattering (GIWAXS) was used to resolve the structural state of CsPbI_3_ PNC samples in the absence and presence of GA.

Figure S3A–D shows the 2D GIWAXS patterns of CsPbI_3_ and Cs_1–*x*_GA_*x*_PbI_3_ PNC films, with the 1D line cuts extracted from out-of-plane directions (see Figure 1B). Attending to the literature and comparing the possible XRD profiles to ascribe the corresponding GIWAXS patterns, we evidenced that the α-cubic phase (ICSD 161481) mostly matches with the 2D diffraction spots. Therefore, all the GIWAXS peaks were associated to 100, 110, 111, 200, 210, and 211 planes at *q* = 1.03, 1.45, 1.77, 2.04, 2.27, and 2.5 Å^–1^, respectively, inferring the presence of the α-cubic phase with high crystallinity,^34,35^ and a preferable orientation relative to the substrate. By focusing on the 1D line cuts of GIWAXS signals associated to 100 and 200 planes (see Figure S3E,E′), we observed a shift to lower *q* values as the GA content increases. This fact indicates the perovskite lattice expansion, ascribed to the incorporation of GA^+^ into the structure.^35,36^ The GIWAXS images in Figure S3 highlight isotropic peak splitting in(*q*_*x*,*y*_)-, i.e., (*q_r_*), and out(*q_z_*)-of-plane. This feature is a signature of a preferential crystallographic behavior in all the samples^36^ which preserves the reference crystal structure of CsPbI_3_ without altering the initial morphology and crystalline phase of PNCs in spite of GA^+^ incorporation.

X-ray photoemission spectroscopy (XPS) was performed to analyze the chemical composition of Cs_1–*x*_GA_*x*_PbI_3_ PNCs. The survey XPS spectra of the materials are presented in Figure S4A, where C, O, Pb, I, and Cs elements were detected. Si was also identified in the PNC samples, possibly coming as an impurity from the initial chemicals used during the PNC synthesis. On the other hand, although N was not directly identified through the survey spectra for composition quantification (possibly due to a low content below that of the detection limit), high-resolution (HR) XPS N 1s spectrum was almost obtained for the qualitative description of chemical speciation (see Figure S4B). Here, we have evidenced a signal at ∼401.8 eV, associated to alkylammonium species. According to the literature, we associated this peak to the presence of GA^+^ species.^33^ Then, although the expected signal from OLAm^+^ (located at 402.8–403 eV for CsPbI_3_ PNCs) was not observed in the N 1s spectrum, we infer its coexistence with GA^+^, considering that OLAm^+^ species are pivotal for nanoparticle stabilization. Figure S5A exhibits HR-XPS Cs 3d spectra of the GA-modified PNCs, where a doublet at 725/739 eV was obtained. These signals are associated to Cs 3d_5/2_ and Cs 3d_3/2_ core levels, indicating the presence of Cs^+^ in the PNC structure.^37^ From the chemical composition estimated for each PNC, summarized in Table S1, a Cs^+^ deficiency is detailed for all the samples, being more evident for the Cs_0.7_GA_0.3_PbI_3_ PNCs. On the other hand, typical 3d_5/2_ and 3d_3/2_ core levels located at ∼619/631 eV appeared in the HR-XPS I 3d spectra of the PNC dispersions (see Figure S5B), associated to the Pb–I bonds from the Cs-(PbI_6_) octahedra.^38^ Here, the Cs_0.9_GA_0.1_PbI_3_ sample presents the lowest iodide deficiency in comparison to other samples (see Table S1).

Then, Figure S5C depicts the HR-XPS Pb 4f spectra of the Cs_1−*x*_GA_*x*_PbI_3_, achieving the characteristic Pb 4f_7/2_ and Pb 4f_5/2_ core levels ∼138/143 eV, respectively. These doublets are ascribed to the existence of Pb^2+^ comprising the nanocrystal lattice.^18^ Considering that PNCs show Cs^+^ and I^–^ surface defects, the materials do not display the existence of undercoordinated lead (or Pb^0^). This is attributed to the washing process of PNCs using methyl acetate (MeOAc), suppressing halide vacancies by introducing COO^–^ anions in the material surface.^39^ Through HR-XPS C 1s and O 1s (see Figure S5D,E), we mainly identified the presence of Si–O, −COOH, and Pb–O bonds. The last two species reinforce the suggestion about the direct interaction between the carboxylate moieties from MeOAc and the surface Pb domains. At this point, by using the atomic percentage of elements measured by XPS and establishing the oxygen fraction associated to the Si–O bonds, we estimated the total oxygen-to-iodine ratio, denoted as O_total_/(I + O_total_). For this calculation, we used the oxygen content coming from COOH groups (associated to carboxylates anchored to the PNC surface) + Pb–O bonds. In this context, the Cs_0.9_GA_0.1_PbI_3_ sample exhibits the lowest oxygen fraction (see Figure 1C), closely followed by Cs_0.8_GA_0.2_PbI_3_. A low oxygen content indicates that less iodide defect sites will be compensated by COO^–^ moieties, obtaining a lesser defective perovskite structure. Then, according to Table S1 and Figure 1C, Cs^+^ deficiency shows a slight increase (lower Cs/(Cs + Pb) ratio) while the GA^+^ addition is increased, and later, a more notable decrease in the Cs/(Cs + Pb) ratio was reached by introducing 30 mol % GA^+^. Accordingly, we can infer that the incorporation of GA at certain quantities (10–20 mol %) can compensate the Cs^+^ defect sites, promoting the introduction of more iodide to passivate halide vacancies. Therefore, it is expected that less oxygen content is required to suppress the halide deficiency. On the contrary, a higher fraction as 30 mol % GA can produce steric hindrance, favoring a more notorious expansion of the crystal structure, as seen by the 1D GIWAX patterns (see Figure S3E,E′), and thereby lattice distortion. This can explain the appearance of a higher density of Cs^+^ and the increase of oxygen fraction to passivate the halide vacancies. On the other hand, by analyzing the PNCs after 180 days of aging, see Table S2, the mixed-cation Cs_0.9_GA_0.1_PbI_3_ sample depicts the lowest decrease of species from its initial composition (mainly iodine), requiring a less amount of oxygen to compensate the halide defects (the lowest O_total_/(I + O_total_)). This fact allows us to deduce that the presence of 10 mol % GA is the most suitable condition to obtain long-term stable and less defective Cs_1–*x*_GA_*x*_PbI_3_ nanoparticles.

Taking into account that GA^+^ incorporation shows an impact on the intrinsic properties of Cs_1–*x*_GA_*x*_PbI_3_ PNCs, it is pivotal to understand how the carrier recombination pathway is affected by the presence of this organic cation during nanocrystal synthesis. Figure 1D shows that for fresh samples, denoted as “0 days,” the presence of GA enhances the PLQY up to ∼97 at 20 mol %. This is a clear indication that the radiative carrier recombination is enhanced, exhibiting the prominent effect of GA^+^.^40^ We infer that after adding 10–20 mol % GA^+^, PNCs with a low density of defect sites are formed, being beneficial to reduce nonradiative traps and producing highly emissive mixed-cation nanocrystals. In accordance with this, fresh GA-modified PNCs show the highest PL intensity by the addition of 10 mol % GA, see Figure 1E. Beyond this cation content, the PL intensity decreases. Then, even the absorption band edge of the samples keeps mostly unchanged with the GA addition, see Figure S6, a slight blue shift in the PL peak position is reached in the presence of GA. In accordance with the observed PL features, we deduce that the presence of 10 mol % GA can compensate Cs^+^ defects into the perovskite during the mixture reaction, opening the door to more iodide species replacing the halide empty sites.^18^ Similar to the formation of oleylammonium iodide which facilitates the stabilization of PNCs during the reaction, we propose the formation of guanidinium iodide to passivate/fill A-site cation defects from the PNC structure during the *instant capping* process. This results in the emergence of smaller nanoparticles with a strengthened quantum confinement, causing the PL displacement to lower wavelengths, and the emergence of a wider band gap for the mixed-cation PNCs. On the contrary, the addition of 30 mol % GA during the PNC synthesis produces enough steric hindrance and lattice strain to avoid an efficient ligand passivation, favoring the preparation of a highly defective PNC product. This can explain the decrease in the Cs^+^ content for the Cs_0.7_GA_0.3_PbI_3_ sample estimated by XPS, see Table S1, deducing the formation of Cs^+^ surface defects (nonradiative carrier traps) to produce a decrease in the PLQY.

Interestingly, after leaving the samples in dark inside a refrigerator for 180 days, all the Cs_1–*x*_GA_*x*_PbI_3_ samples increased their corresponding PLQY values up to 100% in comparison to that of pristine CsPbI_3_ which diminished from 79 to 30%. In addition, slight displacements of the respective PL peak positions were observed for the aged CsPbI_3_ and Cs_0.7_GA_0.3_PbI_3_ samples, see Figure 1E, showing in the first case a blue shift as a consequence of the eventual material degradation, while a red shift was noted in GA-modified PNCs, ascribed to the formation of bigger nanoparticles. In all the cases, FWHM of PNCs was higher after the aging process, as the result of some nanoparticle agglomeration, see Table S3, but the PL intensity of the nanoparticles is almost kept after the aging process. The enhancement of the intrinsic features of the PNCs can be explained by comparing the chemical compositions estimated for the fresh and aged samples, see Tables S1 and S2, respectively. Although a lower iodine content was observed in aged PNCs, the oxygen fraction was also increased, the highest contents being found for Cs_0.8_GA_0.2_PbI_3_ and Cs_0.7_GA_0.3_PbI_3_ samples. Simultaneously, aged pristine CsPbI_3_ and Cs_0.9_GA_0.1_PbI_3_ PNCs exhibit similar Cs contents, but the abrupt discrepancy in their corresponding PLQY allows to infer that the presence of GA compensates the A-site cation deficiency into the nanoparticles. In this context, the higher the GA content added during the PNC synthesis, the passivation of Cs_0.8_GA_0.2_PbI_3_ and Cs_0.7_GA_0.3_PbI_3_ nanoparticles with notorious Cs^+^ deficiency will be favored, providing better photophysical features along the time. Therefore, we hypothesize that the addition of GA can compensate the progressive appearance of defect sites along the time and further enhance the material stability, allowing the PNCs aged for 180 days to present an impressive 100% PLQY. At this stage, we claim that the most suitable condition to obtain high-quality mixed-cation PNCs is by adding 10 mol % GA, where a low density of Cs and I deficiency is achieved to obtain an enhanced optical performance.

On the other hand, to analyze the recombination dynamics of Cs_1–*x*_GA_*x*_PbI_3_ by varying the GA^+^ content, we conducted time-resolved PL (TRPL) measurements on 0 and 180 days’ samples, see Figure S7. Each TRPL curve was fitted by a bi-exponential equation , with the aim to estimate the corresponding average carrier lifetimes, τ_avg_.^33^ Even for PNCs with 100% PLQY, the bi-exponential fitting describes better their PL dynamics. This is associated with the carrier trapping–de-trapping phenomenon taking place in the shallow energy states close to the perovskite conduction band, which is reported in previous studies.^18,41^Tables S4 and S5 summarize the parameters extracted from each TRPL curve. For both fresh and aged samples, we obtained the longest τ_avg_ with 10 mol % GA. τ_avg_ presents an opposite trend to that observed for the oxygen fraction estimated by XPS, see Figure 1C. In this context, by filling/replacing the halide defect sites through oxygen passivation, the emergence of shallow empty O 2p states is reported near the valence band of the perovskite.^42,43^ Therefore, the lower the oxygen fraction into the perovskite, the faster the radiative carrier recombination, pointing out the key role of halide vacancy defects on the PNC surface.

**Figure 2:**
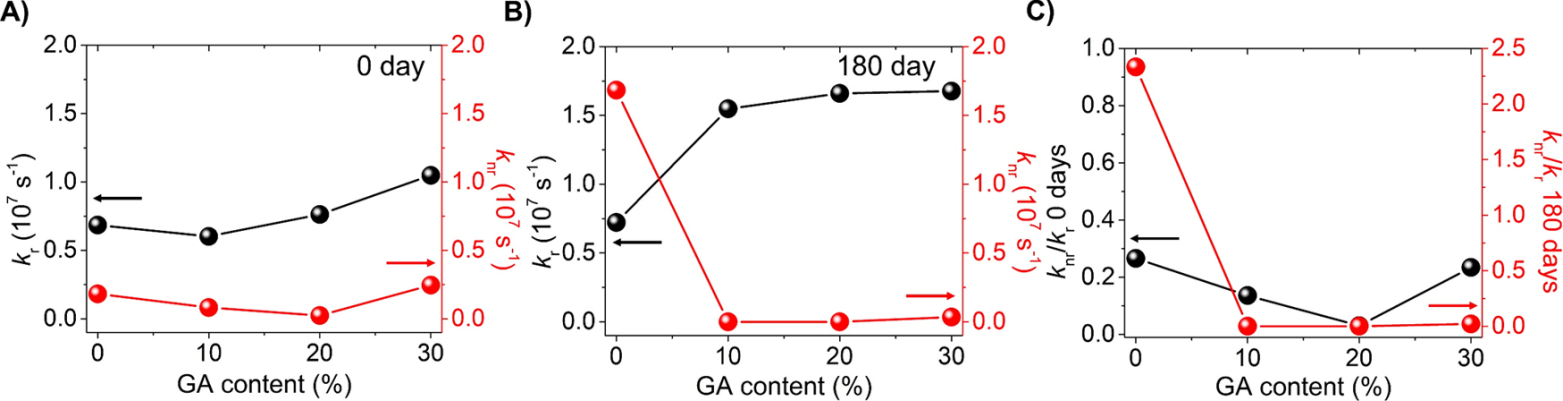
**(**A) Radiative constant (*k*_r_), (B) nonradiative constant (*k*_nr_), and the corresponding (C) *k*_nr_/*k*_r_ of fresh pristine CsPbI_3_ and mixed-cation Cs_1–*x*_GA_*x*_PbI_3_ PNCs (0 day) and aged samples (180 days).

Going deeper into the influence of GA^+^ on the carrier recombination mechanism of Cs_1–*x*_GA_*x*_PbI_3_ PNCs, we have calculated both the radiative and nonradiative recombination constants, *k*_r_ and *k*_nr_, respectively, through the PLQY and τ_avg_ values,^44^ see Tables S4 and S5, Supporting Information, for further details about the calculation methodology. An increase in *k*_r_ and a decrease in the *k*_nr_ and *k*_nr_/*k*_r_ ratios are observed, see Figure 2A–C, respectively, at the higher amounts of GA^+^. This behavior can be more clearly observed in the PNCs after aging them for 180 days, as expected from the higher PLQY of aged samples, see Figure 1D. At this stage, we can corroborate that GA^+^ addition can extend the suppression of nonradiative recombination traps over time, improving the optical performance of the mixed-cation nanocrystals. According to few reports, GA^+^ addition resides on the formation of new hydrogen bonds between the amino groups contained in the organic cation and halide anions on the PNC surface with an AX termination.^40,45^ In this way, a stronger binding to PNCs is triggered, which can explain the prolonged durability of the crystalline phase and photophysical properties of the materials. Thus, through the morphological, structural, and chemical environments and optical measurements, we conclude that the addition of a low GA^+^ content is a promissory approach to produce high-quality iodide-based mixed-cation PNC colloidal solutions without modifying the crystal structure, or optical features, being very useful to prepare PNC active layers and fabricate efficient optoelectronic devices such as LEDs.

**Figure 3:**
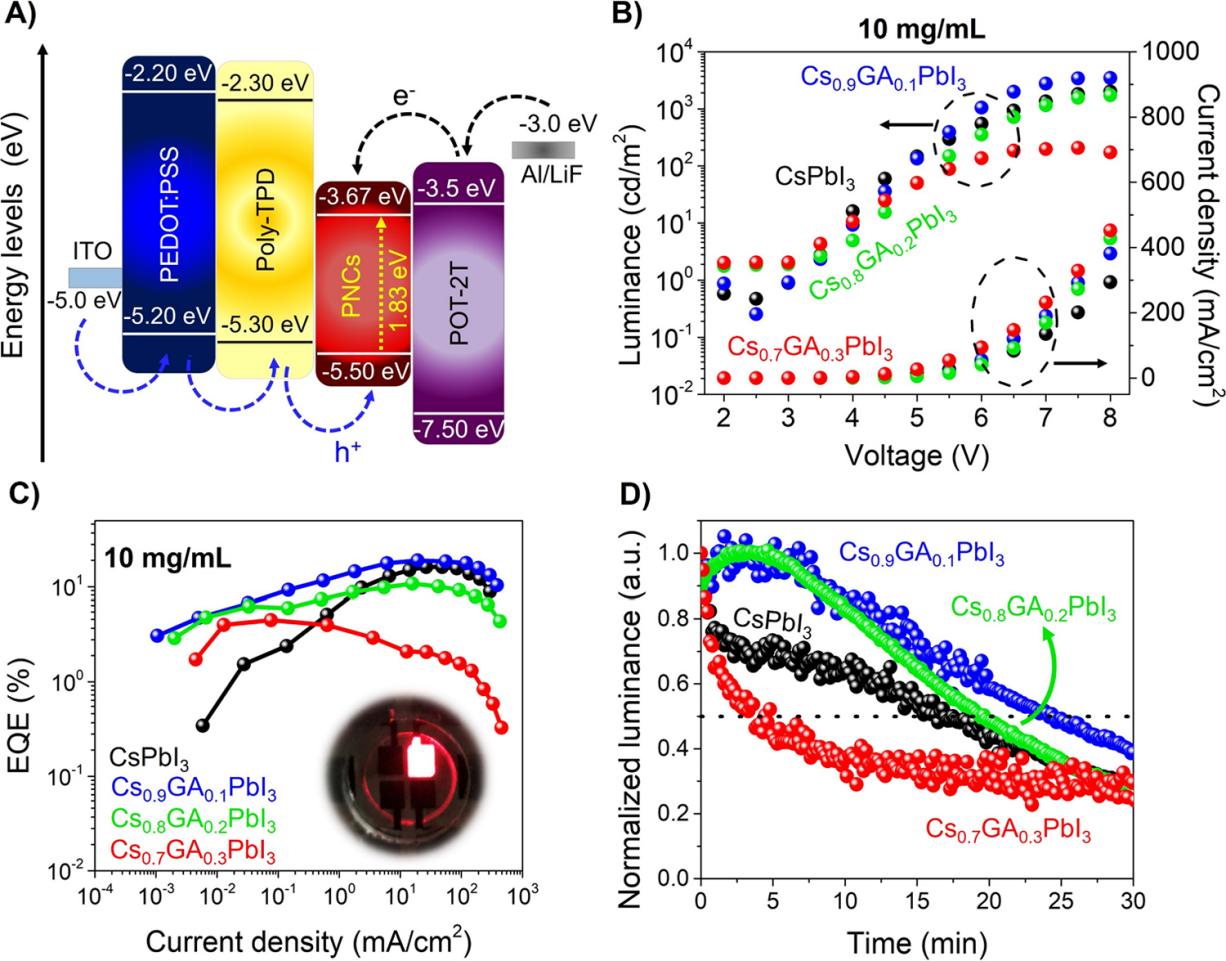
(A) Energy-level diagrams of each component of LED devices. (B) *J*–*V*–*L* and (C) EQE vs *J* curves of mixed-cation perovskites at 10 mg/mL. Inset of Figure 3C shows a R-LED under operation. (D) Stability measurement of encapsulated devices at constant 5 V for 30 min (equivalent to 80–100 cd/m^2^, except for GA_0.3_ of 50 cd/m^2^).

Having observed the enhancement of PL properties of the mixed-cation Cs_1–*x*_GA_*x*_PbI_3_ PNCs by the presence of GA, especially after aging, we introduced these materials as an emissive layer for the fabrication of perovskite R-LEDs. To implement this application, devices possessed the following architecture:^46^ PEDOT:PSS/Poly-TPD/PNCs/PO-T2T/LiF/Al (see Figure 3A; see Supporting Information for more details). Relative band positions exhibited for this perovskite R-LED configuration have been reported earlier,^18^ which ensures the efficient carrier injection into the active layer.

The deposition of the active layer was optimized by varying the PNC concentration for thin-film deposition. We used pristine CsPbI_3_ PNCs at six different concentrations: 1, 5, 10, 20, 30, and 40 mg/mL. Parameters obtained for each concentration such as current density (*J*), luminance (*L*), and EQE are summarized in Tables S6–S8 and shown in Figure S8A and S8B, observing the maximum performance for a PNC concentration of 10 mg/mL. A higher concentration of nanocrystals could induce the material agglomeration, which can hinder the carrier injection to reach the emissive layer.^47^

Then, we prepared multiple batches of LEDs using the mixed-cation PNCs by varying the GA^+^ content for the optimized concentration of 10 mg/mL. Figure 3B,C illustrates the LED performance of Cs_0.9_GA_0.1_PbI_3_ (*L* = 3486 cd/m^2^ and EQE = 18.9%), Cs_0.8_GA_0.2_PbI_3_ (*L* = 1732 cd/m^2^ and EQE = 10.8%), and Cs_0.7_GA_0.3_PbI_3_ (*L* = 173 cd/m^2^, and EQE = 4.5%). All the devices exhibit a turn-on voltage (*V*_ON_) around 3 V. Considering that the highest EQE was obtained by using mixed-cation PNCs, in particular for 10 mol % GA addition, this cation content is the most adequate condition for LED devices. In accordance with the characterization performed, the presence of the richest surface of I^–^, i.e., lower O_total_/(I + O_total_) ratio, and the longer lifetime decay obtained in TRPL favors the carrier transport into the device at low energy. This fact can be detailed in the electroluminescence (EL) spectra acquired at 675 nm (deep red color beyond the Rec 2020 standards) for Cs_1–*x*_GA_*x*_PbI_3_, achieving the highest EL intensity at 10 mol % GA, Figure S9. Concerning the device stability, Figure 3D displays the normalized luminance as a function of time, where the R-LEDs were operating at 5 V, equivalent to 80–100 cd/m^2^, except for GA_0.3_ where the luminance was 50 cd/m^2^ due to the lower performance of the device. We determined the operational half-life (*t*_50_) from each material composition, finding values around 15, 25, 20, and 4 min for pristine CsPbI_3_, Cs_0.9_GA_0.1_PbI_3_, Cs_0.8_GA_0.2_PbI_3_, and Cs_0.7_GA_0.3_PbI_3_, respectively. Hence, we can claim that a GA^+^ content around 10 mol % is suitable to delaying the deterioration of the optical properties and the efficiency of the devices. On the other hand, the operational parameters for the Cs_0.9_GA_0.1_PbI_3_ R-LED device described above are higher than the performance of R-LEDs already reported, where the use of purified PbI_2_ precursors was introduced to obtain high-quality PNC active layers (*V*_ON_ = 5 V, *L* = 832 cd/m^2^, *J* = 260 mA/cm^2^, and EQE = 13.6%). Then, although operational stability was not shown in the previous contribution, we can suggest that the GA-based R-LED device shows a longer stability, considering that the roll-off effect starts to occur at currents higher than 100 mAcm^–2^, compared to the reported device. The above advancements are an indication that the carrier injection was enhanced into the R-LED in the presence of GA to favor the radiative recombination process. Therefore, we exhibit that the incorporation of GA^+^ into the mixture reaction to partly replace Cs^+^ from the perovskite structure can improve the intrinsic features and stability of red-emitting PNC colloidal solutions, giving an insight about the A-site cation tailoring for achieving more efficient optoelectronic devices.

## 3 Conclusions

In this work, we have shown the introduction of GA^+^ cations into the mixture reaction, with the purpose to synthesize high-quality mixed-cation Cs_1–*x*_GA_*x*_PbI_3_ PNCs through in situ synthesis. The presence of the big cation does not alter the morphology and the crystalline phase of these materials, improving the optical performance of the PNC colloidal solutions. We elucidated that GA^+^ promotes the compensation of surface defects such as Cs^+^ and I^–^ vacancies, which allows to obtain nanocrystals with long-term stability stored in the fridge, with PLQY up to 100% after 180 days of PNC synthesis. Here, 10 mol % GA^+^ is adequate to suppress the nonradiative recombination dynamics, giving the possibility to favor the radiative carrier recombination into the photomaterial. Interestingly, this defect compensation is maintained over time, delaying the deterioration of the photophysical features of the Cs_1–*x*_GA_*x*_PbI_3_ PNCs. Under this premise, we were able to fabricate bright R-LEDs with a maximum EQE ∼ 19%, and even more important with a higher stability, presenting *t*_50_ = 25 min, increasing 67% with respect to R-LEDs prepared with CsPbI_3_ PNCs. Thus, as it is expected, the lowered defect mixed-cation nanocrystals trigger an effective carrier injection through the device, improving the operational performance and stability. An excess of GA^+^ during the PNC synthesis induces a steric hindrance in the material surface, restraining the defect passivation, promoting the eventual loss of the intrinsic properties of the nanocrystals, and thereby the decrease of LED efficiency. This paper provides a new direction for achieving phase-stabilized red-emitting PNCs suitable for the development of promising LED technologies.

## 4 Experimental Section

### 4.1 Materials

All materials were reagent grade and were used as received. Lead iodide (PbI_2_, >98%, from TCI), cesium carbonate(Cs_2_CO_3_, >99%, from Sigma Aldrich), guanidinium acetate salt (GAOAc, >99%, from Sigma Aldrich), oleic acid (OA, technical grade 90%), oleylamine (OLA, technical grade 98% from Sigma Aldrich), 1-octadecene (1-ODE, technical grade 90% from Sigma Aldrich), hexane (reagent grade 97%), methyl acetate (MeOAc, anhydrous 99.5% from Sigma Aldrich), 2-propanol (99.7% from Carlo Erba), ethanol (96%) and acetone (99.25%) (from PanReac), hydrochloric acid (HCl 37%), zinc powder (99.995%), PEDOT:PSS (Clevios 4083from Heraeus), Al, lithium fluoride (LiF, from Lumtec), PO-T2T (from Lumtec), and poly-TPD (from Lumtec) were used. EPOXY encapsulation (from Lumtec) and indium tin oxide (ITO)-coated glass substrates (Pilkington TEC15, ∼15 Ω sq^–1^) were also used.

### 4.2 Synthesis and Purification of CsPbI_3_ PNCs

To synthesize CsPbI_3_ PNCs, 0.407 g of Cs_2_CO_3_ (1.25 × 10^–3^ mol), 1.25 mL (OA) and 20 mL of 1-ODE were mixed in a 25 mL three-necked flask and maintained at 120 °C under vacuum for 60 min with constant stirring. After 60 min, the temperature was increased to 150 °C under N_2_ purge to complete the dissolution of Cs_2_CO_3_. Then, the temperature was decreased to the previous value of 120 °C to avoid Cs-oleate oxidation. In parallel, 0.5 g of PbI_2_ and 25 mL of 1-ODE were loaded together in a 100 mL three-necked flask (reaction flask) and kept under vacuum at 120 °C for 30 min. After 30 min, N_2_ was filled in the reaction flask, and 2.5 mL of OA and OLA ligands were added to it. Once the mixture became transparent, it was left for another 30 min. After 1 h of ligand treatment, the reaction flask temperature was increased up to 170 °C, and 2.5 mL of preheated Cs-oleate was quickly injected and moved to an ice bath where it was immersed for rapid cooling to the ambient temperature. The as-prepared crude product was centrifuged at 5000 rpm for 5 min. The supernatant was discarded, and the precipitate of PNCs was recovered by redispersing it in 5 mL of anhydrous hexane and 5 mL of MeOAc. Again, the PNC dispersion was centrifuged at 5000 rpm for 5 min, and the supernatant was discarded, and the precipitate was redispersed with 1 mL of anhydrous hexane. Finally, the solutions of CsPbI_3_ PNCs were stored for 24 h in a refrigerator (5 °C) prior to use (in order to precipitate excess of Cs-oleate and Pb-oleate).

### 4.3 Synthesis of Cs_1–*x*_GA_*x*_PbI_3_ PNCs

The synthesis of the mixed-cation Cs_1–*x*_GA_*x*_PbI_3_ PNCs was performed by following the abovementioned procedure for pristine CsPbI_3_, with some modifications in the preparation of the Cs^+^/GA^+^ solutions. Here, different percentages of the guanidinium acetate (GAOAc) precursor, around 10, 20, and 30 mol %, were mixed with the corresponding amount of Cs_2_CO_3_ to complete the synthesis of the total 1.25 × 10^–3^ mol of the A-site cation. In this way, the above three solutions were prepared by mixing Cs_2_CO_3_/GAOAc = (0.37 g/0.03 g), (0.33 g/0.06 g), and (0.29 g/0.09 g), respectively.

### 4.4 LED Fabrication

ITO prepatterned substrates were cleaned with deionized water, acetone, and ethanol in an ultrasonic cleaner for 15 min for each solvent. After being dried by air flow, the substrates were put in an UV–ozone system for 15 min to remove organic residues. Once cleaned, the hole transport layer (HTL) (PEDOT:PSS) was filtered with a 0.45 μm PVDF filter and deposited. Then, it was spin-coated onto the ITO substrates with an acceleration of 3000 rpm for 30 s and heated at 130 °C for 15 min. After the HTL deposition, the substrates were moved to a N_2_-filled glovebox to deposit 100 μL of poly-TPD of 6 mgmL^–1^ over PEDOT:PSSHTL by a one-step spin coating at 3000 rpm for 30 s. Then, the substrates were moved to a hot plate and annealed for 10 min at 100 °C. Following these steps, 100 μL of the perovskite QDs in hexane with different concentrations was deposited at 3000 rpm for 30 s. After the perovskite layer deposition, the electron transport layer (ETL) was deposited by the thermal evaporation of 40 nm of PO-T2T, continued by 1 nm of LiF. Finally, 100 nm of Al metal contact is deposited by thermal evaporation. Then, the different parameters of devices (EL, luminance, current density, and EQE) were obtained by employing a Hamamatsu EQE measurement system (C9920-12) coupled with an integrating sphere connected to a Keithley 2400 instrument as a source meter of current/voltage and a PMA-12 photonic multichannel detector. Each of the devices was encapsulated and measured from 2 to 8 V at a step of 0.5 V. Decay measurements were made by setting 5 V continuously and measuring Ld every 10 s for 30 min.

### 4.5 Optical Characterization

UV–vis absorption of the thin films was measured using a UV–vis absorption spectrophotometer (Varian, Cary 300) in the wavelength range of 400–850 nm. Steady-state photoluminescence emission (PL) and time-resolved photoluminescence (TRPL) were measured using a PL spectrophotometer (Fluorolog 3-11, Horiba) using 400 nm excitation wavelength for PL and TRPL at the wavelength corresponding to the maximum intensity by employing a pulsed laser (NanoLED-405L, <100 ps of pulse width, 1 MHz frequency). Photoluminescence quantum yield (PLQY) was measured using a Hamamatsu PLQY absolute QY measurement system C9920-02, coupled to an integrating sphere, at an excitation wavelength of 400 nm. The obtained values were adjusted in an absorbance range around 0.5 to conduct the measurements, these values being suitable to achieve the maximum PLQY in the samples.

### 4.6 Structural Characterization

TEM is performed by employing a field emission tunneling electron microscope (Hitachi HF-3300), with an applied bias of 300 kV. Also, the SAED patterns of the crystalline structure were obtained, and the particle size was obtained from the TEM images using ImageJ software to measure them.

#### 4.6.1 Grazing Incidence Wide Angle X-ray Scattering

All measurements have been performed in air at the BL9 beamline of DELTA synchrotron. Single-shot images (100 s exposition) were recorded at room temperature using a MAR area detector with a wavelength of 0.82657 Å (15 keV incident energy), a sample-to-detector distance of 495 mm (calibrated with a LaB_6_ reference sample), and at an angle of incidence of 0.15° with a beam size of 1 × 0.2 mm^2^. The data were processed and analyzed using FIT2D software.

#### 4.6.2 X-ray Photoelectron Spectroscopy

To analyze the chemical composition at the surface, XPS was performed using an XPS system (ESCA-2R, Scienta-Omicron). Here, PNC colloidal solutions were deposited by drop-casting on Ti sheets heated at 50 °C, under ambient conditions, obtaining the corresponding PNC layers. The spectra were recorded at an energy of 50 eV for survey and HR, respectively, by employing monochromatic Al Kα = 1486.6 eV. It was possible to recognize the elements C 1s, Pb 4f, I 3d, O 1s, N 1s, and C 1s. Si impurity was also detected. Then, adventitious carbon (284.8 eV) was utilized for reference at the BE scale. To analyze the data, CasaXPS processing software (Casa software Ltd.) was used.

### 4.7 Constant Calculations of Adjusted TRPL Curves^44^

The decay curves were fitted according to the following equation:
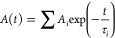


Also, the average lifetimes(τ_avg_) of curves were obtained using
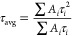


To find the luminescence response of the material to obtain the radiative and nonradiative values, the average lifetime and PLQY were needed, expressed in the range of 0–1, employing the following equations:
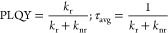


At the end, it is possible to obtain the radiative recombination rate (*k*_r_) and the nonradiative recombination rate (*k*_nr_):
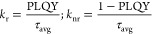


## Supporting Information Available

Experimental setup, histograms, SAED measurements, 2D GIWAX rings, UV–vis absorption, XPS analysis, surface chemical composition and time-resolved PL decay measurements of mixed-cation Cs_1–*x*_GA_*x*_PbI_3_ PNCs, and CsPbI_3_-based LED performance by varying the perovskite concentration and electroluminescence spectra of the mixed-cation nanocrystals in the presence and absence of GA (PDF)
