# Supplementary material for: Increasing the Performance and Stability of Red-Light-Emitting Diodes Using Guanidinium Mixed-Cation Perovskite Nanocrystals
Source: Chem Mater. 2023 May 9;35(10):3998–4006. doi: 10.1021/acs.chemmater.3c00269 (PMC10210241; doi:10.1021/acs.chemmater.3c00269)
Supplement: Supplementary file 1 — cm3c00269_si_001.pdf [file cm3c00269_si_001.pdf]

Supporting Information

Increasing Performance and Stability of Red-Light Emitting Diodes Using Guanidinium Mixed-Cation Perovskite Nanocrystals

Patricio Serafini,[1] Alexis Villanueva-Antolí,[1] Samrat Das Adhikari,[1] Sofia Masi,[1] Rafael S. Sánchez,[1] Jhonatan Rodriguez-Pereira,[2,3] Bapi Pradhan,[4] Johan Hofkens,[4] Andrés F. Gualdrón-Reyes,[1,5] and Iván Mora-Seró[1]**

[1]Institute of Advanced Materials (INAM), Universitat Jaume I, Avenida de Vicent Sos Baynat, s/n, 12071 Castelló de la Plana, Castellón, Spain.

[2] Center of Materials and Nanotechnologies, Faculty of Chemical Technology, University of Pardubice, 53002 Pardubice, Czech Republic.

[3]Central European Institute of Technology, Brno University of Technology, 612 00 Brno, Czech Republic.

[4]Laboratory for Photochemistry and Spectroscopy, Molecular Imaging and Photonics, Department of Chemistry, Katholieke Universiteit Leuven, Celestijnenlaan 200F – bus 2404 B-3001 Heverlee, Belgium.

[5]Facultad de Ciencias, Instituto de Ciencias Químicas, Isla Teja, Universidad Austral de Chile, 5090000, Valdivia, Chile.

Corresponding authors: gualdron@uji.es; sero@uji.es

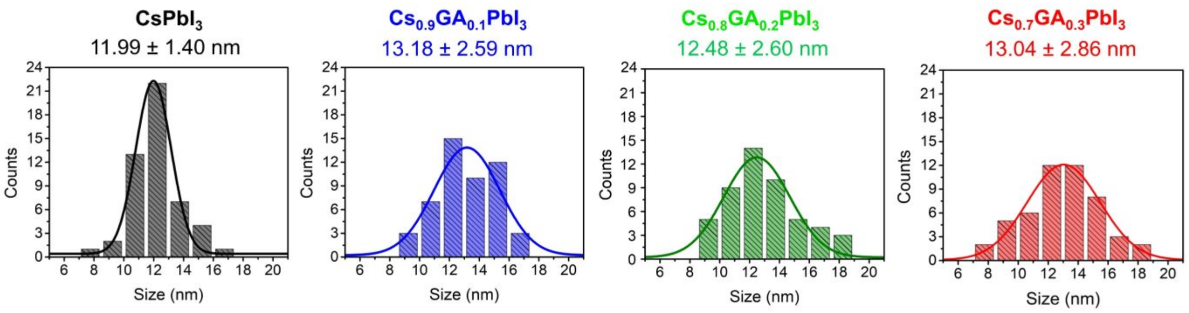

Figure S1. Particle size distribution obtained from TEM measurements of pristine $CsPbI_3$ and mixed-cation $Cs_{1-x}GA_xPbI_3$ PNCs.

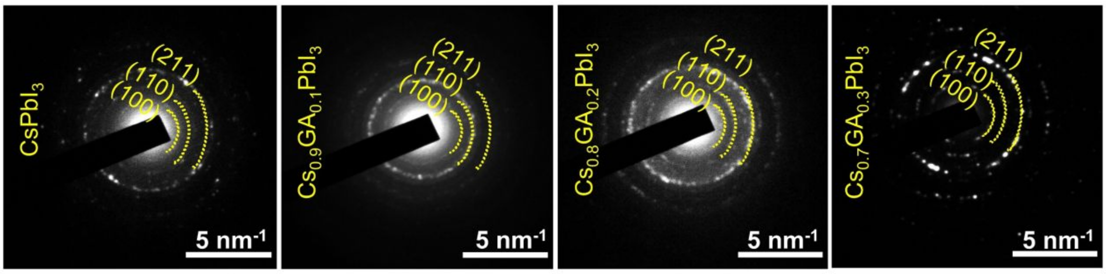

Figure S2. SAED measurements of pristine $CsPbI_3$ and mixed-cation $Cs_{1-x}GA_xPbI_3$ PNCs.

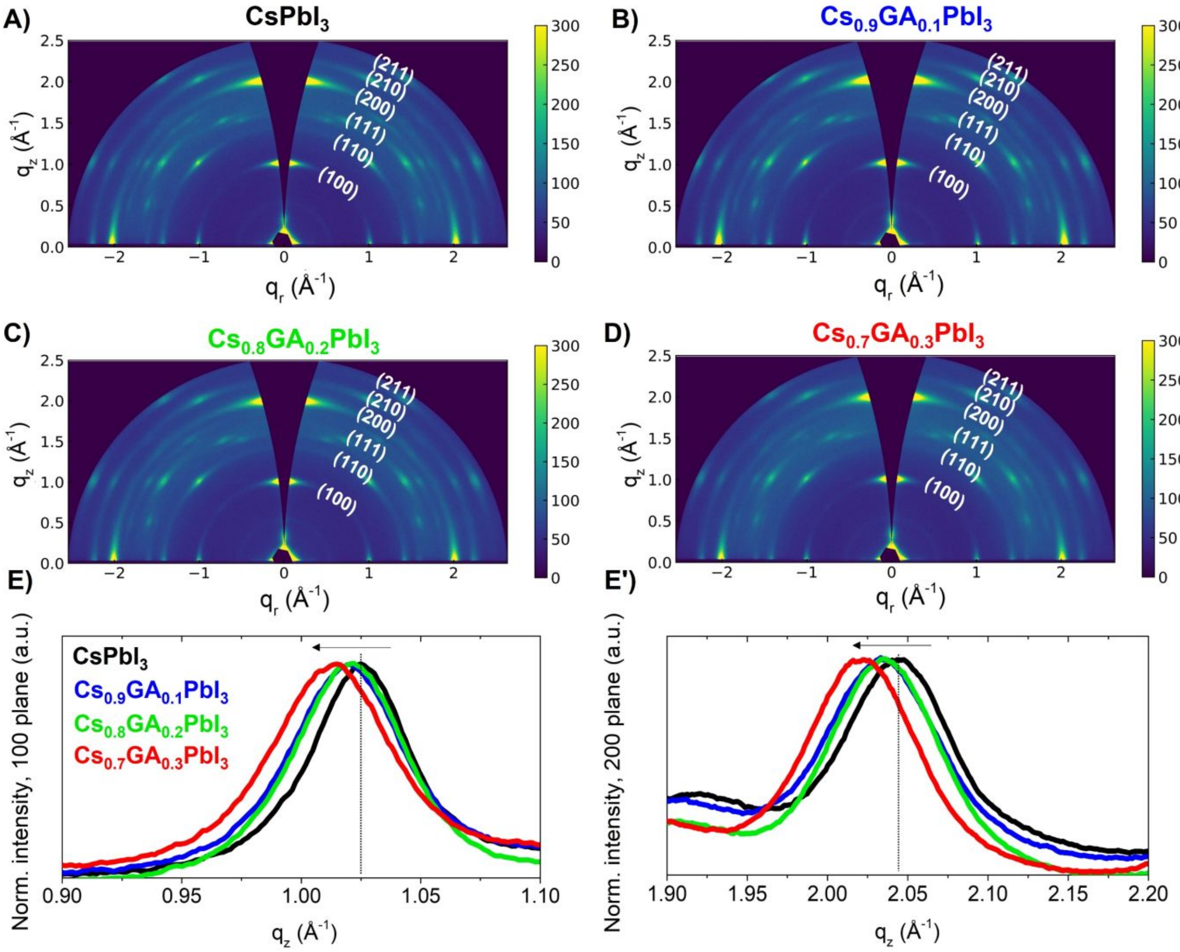

Figure S3. A-D) 2D GIWAXS rings of 1 layer of pristine $CsPbI_3$ and mixed-cation $Cs_{1-x}GA_xPbI_3$ PNCs film. Normalized intensity of the 1D GIWAXS peaks associated with the E) 100 and E') 200 planes.

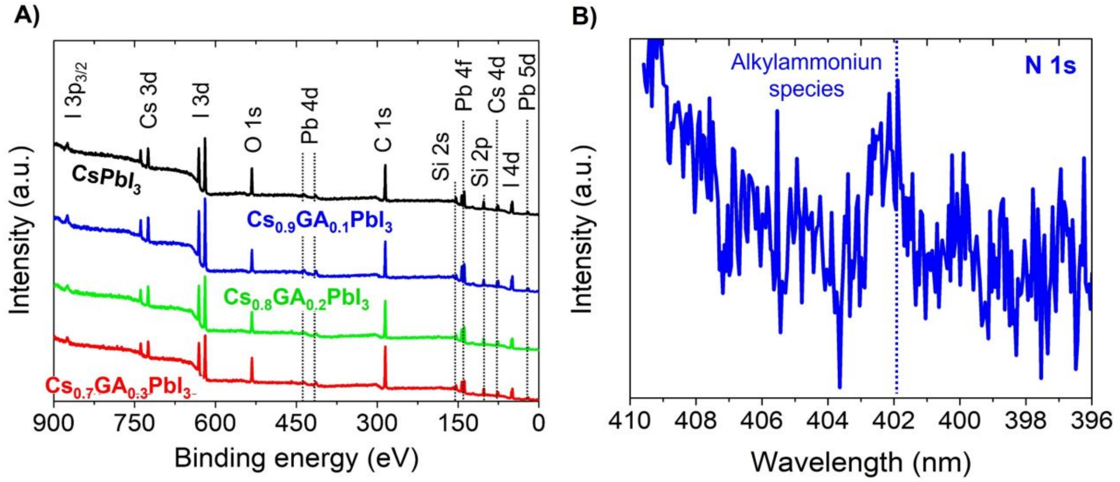

Figure S4. A) XPS survey spectra of pristine $CsPbI_3$ and mixed-cation $Cs_{1-x}GA_xPbI_3$ PNCs with different GA content incorporated perovskite PNCs. B) HR-XPS N 1s spectrum of $Cs_{0.9}GA_{0.1}PbI_3$ PNCs sample.

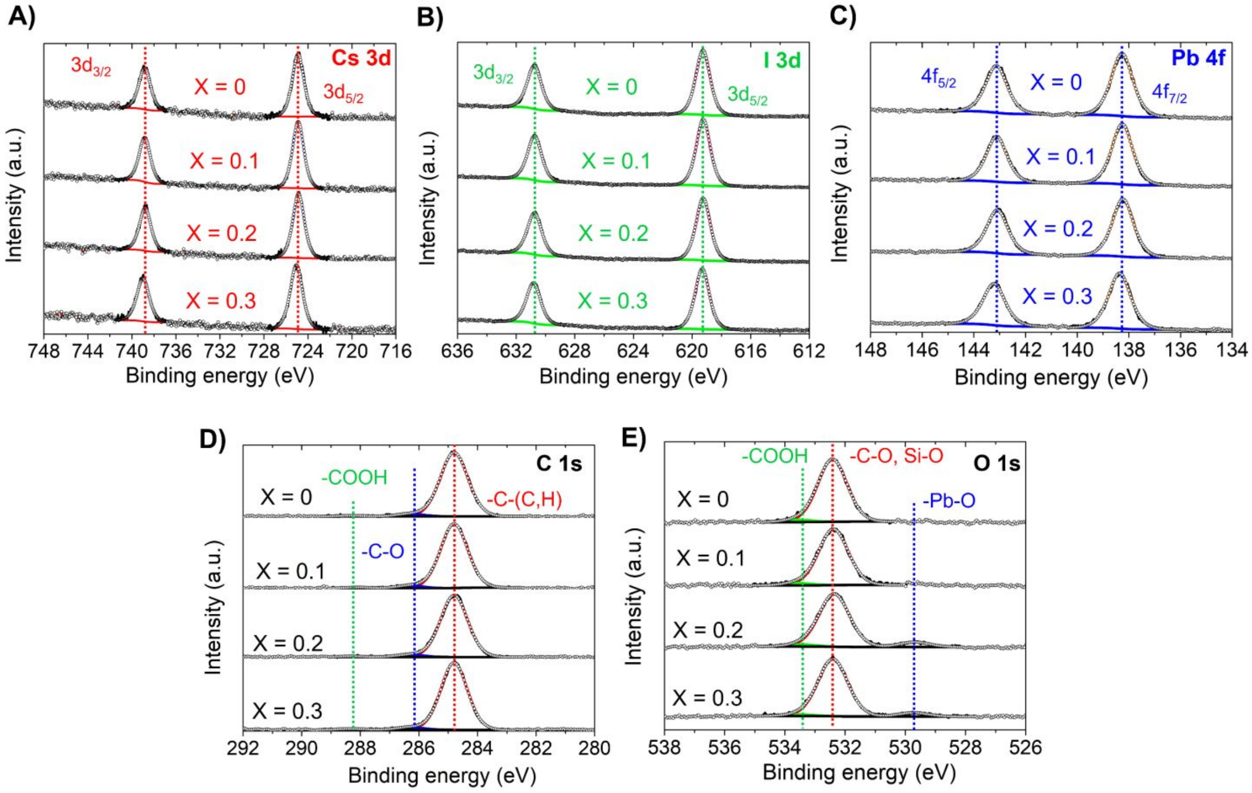

Figure S5. HR-XPS spectra of main elements: A) Cs 3d, B) I 3d, C) Pb 4f, D) C 1s and E) O 1s respectively of pristine $CsPbI_3$ and mixed-cation $Cs_{1-x}GA_xPbI_3$ PNCs.

Table S1. Chemical atomic composition of fresh pristine $CsPbI_3$ and mixed-cation $Cs_{1-x}GA_xPbI_3$ PNCs (0 day) obtained from XPS analysis.

PNCs sample	Atomic concentration [%]										Relative concentrations			Ratios with COOH + Pb-O	
	C-(C,H)	C-O	COOH	Si-O, C-O	Pb-O	COOH	I^-	Pb^{2+}	Cs^+	Si-O	Cs	Pb	I	O/(I+O)	Cs/(Cs+Pb)
X=0	57.74	2.41	0.82	15.19	-	0.58	4.90	1.82	1.51	15.05	0.83	1.00	2.69	0.11	0.46
X= 0.1	60.21	2.78	1.10	12.74	-	0.54	6.99	2.54	2.13	10.96	0.84	1.00	2.75	0.07	0.45
X =0.2	60.74	2.51	0.85	13.21	1.23	0.52	5.16	1.98	1.62	12.17	0.82	1.00	2.61	0.25	0.45
X =0.3	62.72	2.51	0.96	13.07	0.85	0.51	4.05	1.61	1.24	12.47	0.77	1.00	2.52	0.25	0.44

Table S2. Chemical atomic composition of aged pristine $CsPbI_3$ and mixed-cation $Cs_{1-x}GA_xPbI_3$ PNCs (180 days) obtained from XPS analysis.

PNCs sample	Atomic concentration [%]										Relative concentrations			Ratios with COOH	
	C-(C,H)	C-O	C=O	COOH	Si-O, C-O	COOH	I^-	Pb^{2+}	Cs^+	Si-O	Cs	Pb	I	O/(I+O)	Cs/(Cs+Pb)
X=0	62.81	2.34	0.68	0.44	14.87	1.24	2.21	0.91	0.69	13.82	0.76	1.00	2.43	0.36	0.43
X= 0.1	64.05	2.44	0.23	0.30	13.07	1.10	3.21	1.30	0.97	13.34	0.75	1.00	2.47	0.26	0.43
X =0.2	61.79	2.22	0.43	0.26	15.06	1.44	1.54	0.68	0.45	16.12	0.66	1.00	2.26	0.48	0.40
X =0.3	66.12	2.21	0.34	0.59	12.36	1.50	2.04	0.84	0.55	13.45	0.65	1.00	2.43	0.42	0.40

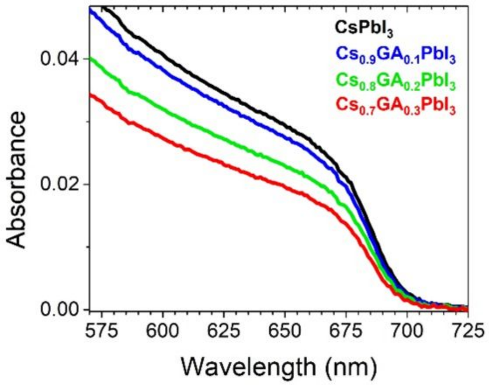

Figure S6. UV-vis spectra of pristine $CsPbI_3$ and mixed-cation $Cs_{1-x}GA_xPbI_3$ PNCs.

Table S3. PL features of fresh pristine $CsPbI_3$ and mixed-cation $Cs_{1-x}GA_xPbI_3$ (0 days) and aged samples (180 days).

Perovskite sample	PL peak position (nm) 0 days	PL peak position (nm) 180 days	FHWM (nm) 0 days	FWHM (nm) 180 days
$CsPbI_3$	693	686	38.0	44.0
$Cs_{0.9}GA_{0.1}PbI_3$	691	690	42.6	46.0
$Cs_{0.8}GA_{0.2}PbI_3$	690	690	40.2	45.3
$Cs_{0.7}GA_{0.3}PbI_3$	688	691	41.7	46.8

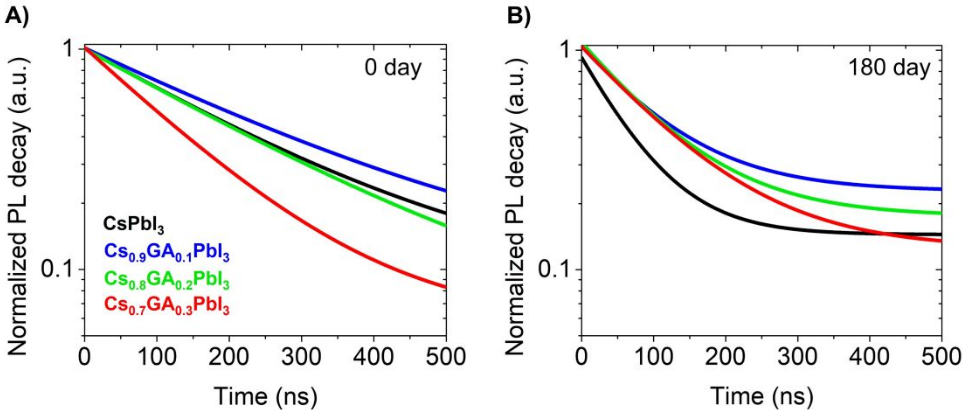

Figure S7. Time resolved photoluminescence (TRPL) of pristine $CsPbI_3$ and mixed-cation $Cs_{1-x}GA_xPbI_3$ PNCs at A) 0 day and B) 180 days.

Table S4. Parameters obtained by fitting the TRPL curve of fresh samples (0 Day).

0 Day	A_1 (%)	τ_1 (ns)	A_2 (%)	τ_2 (ns)	PLQY	τ_{avg} (ns)	k_r (10^7 s^{-1})	k_{nr} (10^7 s^{-1})	k_{nr}/k_r
$CsPbI_3$	0.83	163.63	0.17	475.83	0.79	115.31	0.68	0.18	0.27
$Cs_{0.9}GA_{0.1}PbI_3$	0.94	180.88	0.06	585.64	0.88	146.92	0.59	0.08	0.14
$Cs_{0.8}GA_{0.2}PbI_3$	0.87	175.61	0.13	493.25	0.97	126.57	0.76	0.02	0.03
$Cs_{0.7}GA_{0.3}PbI_3$	0.85	109.51	0.15	294.46	0.81	77.34	1.05	0.25	0.23

Table S5. Parameters obtained by fitting the TRPL curve of aged samples (180 Day).

180 Day	A_1 (%)	τ_1 (ns)	A_2 (%)	τ_2 (ns)	PLQY	τ_{avg} (ns)	k_r (10^7 s^{-1})	k_{nr} (10^7 s^{-1})	k_{nr}/k_r
$CsPbI_3$	0.92	54.55	0.08	223.86	0.30	41.58	0.72	1.68	2.33
$Cs_{0.9}GA_{0.1}PbI_3$	0.91	85.65	0.09	303.48	1.00	64.60	1.55	0.00	0.00
$Cs_{0.8}GA_{0.2}PbI_3$	0.89	81.18	0.11	259.99	1.00	60.23	1.66	0.00	0.00
$Cs_{0.7}GA_{0.3}PbI_3$	0.81	80.91	0.19	257.08	0.98	58.46	1.68	0.03	0.02

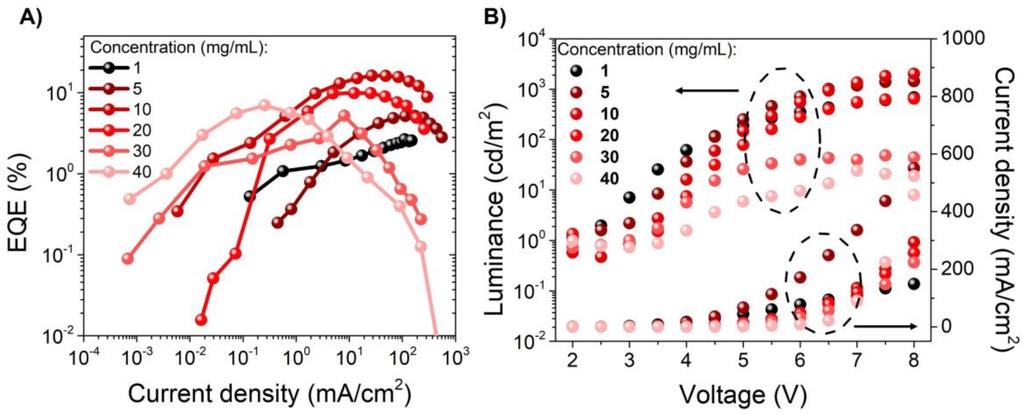

Figure S8. A) EQE vs Current density (J) and B) J-V-L curves of the devices made with $CsPbI_3$ PNCs with different concentration from 1 mg/mL to 40 mg/mL.

Table S6. Current density of $CsPbI_3$-based LEDs prepared with PNCs concentrations from 5 to 40 mg/mL.

$CsPbI_3$	Current density (mA/cm^2)					
	Concentration (mg/mL)					
**Voltage (V)**	***1***	***5***	***10***	***20***	***30***	***40***
**2.0**	0.13	0.45	0.01	0.47	>0.01	>0.01
**2.5**	0.56	0.81	0.03	0.64	>0.01	>0.01
**3.0**	2.98	1.84	0.14	0.89	0.02	0.02
**3.5**	8.45	5.08	0.63	0.32	0.15	0.08
**4.0**	17.39	15.06	2.31	1.65	0.79	0.26
**4.5**	29.59	35.60	6.62	4.93	2.72	0.78
**5.0**	44.49	67.30	14.10	12.37	8.06	1.74
**5.5**	60.67	113.48	26.52	25.31	17.10	3.91
**6.0**	77.91	171.54	48.42	47.24	32.76	9.20
**6.5**	94.18	248.53	83.86	80.13	56.50	22.04
**7.0**	110.48	335.78	135.11	120.14	92.38	89.9
**7.5**	133.86	436.95	201.43	183.94	148.09	224.35
**8.0**	149.11	551.00	293.80	256.87	223.79	524.05

Table S7. Luminance of $CsPbI_3$-based LEDs prepared with PNCs concentrations from 5 to 40 mg/mL.

$CsPbI_3$	Luminance (cd/m^2)					
	Concentration (mg/mL)					
**Voltage (V)**	***1***	***5***	***10***	***20***	***30***	***40***
**2.0**	1.06	1.16	0.58	0.35	0.72	0.68
**2.5**	1.96	1.60	0.47	0.81	0.81	0.73
**3.0**	7.09	2.20	0.89	0.85	1.00	0.78
**3.5**	25.51	8.50	2.74	1.52	1.75	0.88
**4.0**	61.95	36.52	16.11	7.48	5.68	1.57
**4.5**	117.20	113.40	59.89	31.59	15.42	3.62
**5.0**	189.00	251.40	147.00	78.35	25.91	5.95
**5.5**	269.70	458.40	297.20	161.60	33.48	7.54
**6.0**	351.60	712.20	551.20	280.00	40.03	9.61
**6.5**	430.94	1009.00	933.70	401.10	43.21	13.51
**7.0**	541.47	1186.00	1353.00	552.10	49.66	24.18
**7.5**	604.16	1394.00	1816.00	622.30	48.43	20.95

Table S8. EQE of $CsPbI_3$-based LEDs prepared with PNCs concentrations from 5 to 40 mg/mL.

$CsPbI_3$	EQE (%)					
	Concentration (mg/mL)					
**Voltage (V)**	*1*	*5*	*10*	*20*	*30*	*40*
**2.0**	0.52	0.25	0.43	0.05	0.36	0.86
**2.5**	1.07	0.36	1.54	0.16	1.34	1.00
**3.0**	1.24	0.79	2.39	0.10	1.25	0.30
**3.5**	1.45	1.84	5.14	2.86	1.53	0.56
**4.0**	1.67	3.12	9.78	5.86	2.26	0.70
**4.5**	1.89	4.20	12.99	9.76	1.48	0.56
**5.0**	2.08	4.86	15.05	9.84	0.86	0.47
**5.5**	2.25	5.14	16.18	9.87	0.57	0.29
**6.0**	2.37	5.18	16.15	8.97	0.43	0.16
**6.5**	2.43	4.90	15.60	7.56	0.36	0.09
**7.0**	2.65	4.14	13.76	6.86	0.31	0.04
**7.5**	2.50	3.59	12.08	4.89	0.23	0.01
**8.0**	2.57	2.81	8.88	3.57	0.15	> 0.01

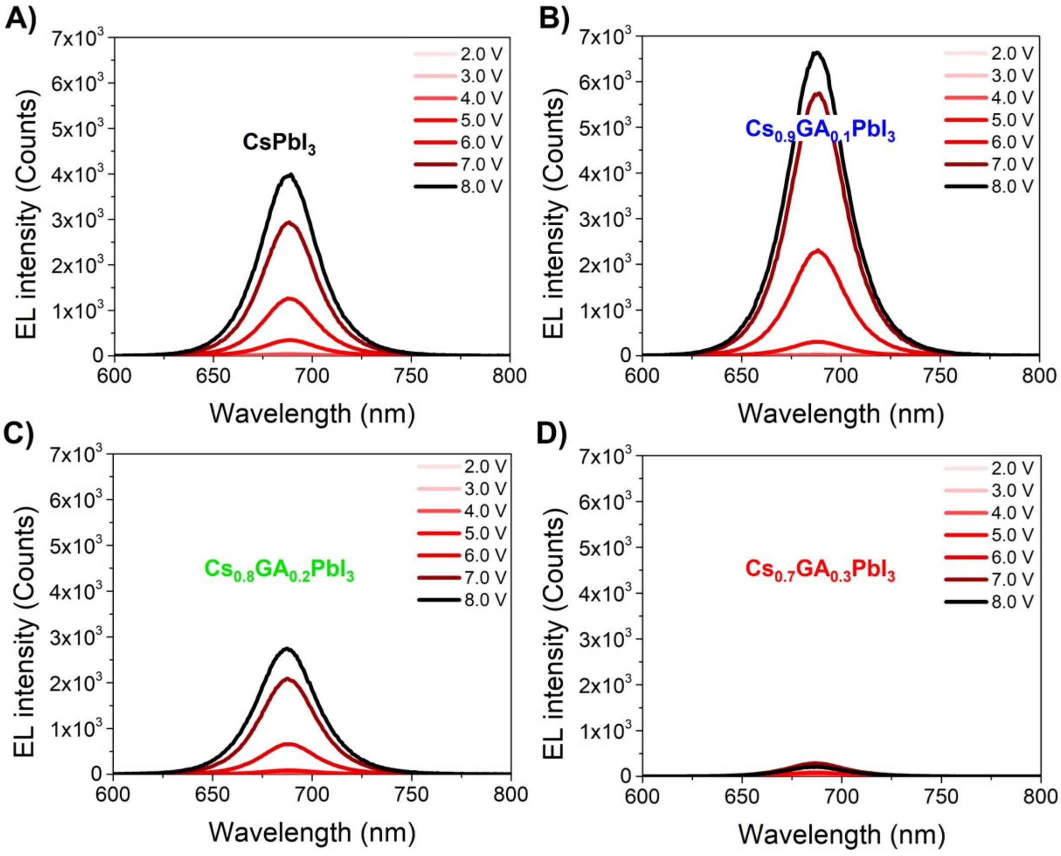

Figure S9. Electroluminescence spectra of R-LEDs based on pristine $CsPbI_3$ and mixed-cation $Cs_{1-x}GA_xPbI_3$ PNCs with concentration of 10 mg/mL.
